# Nanoparticle-based drug delivery systems targeting inflammatory immune mechanisms in acute myocardial infarction: current advances and perspectives

**DOI:** 10.3389/fcvm.2025.1657300

**Published:** 2025-10-23

**Authors:** Yuxuan Li, Peng Li, Wujiao Wang, Yuanyuan Zhang, Sinai Li, Dong Li, Qian Lin

**Affiliations:** ^1^ Department of Cardiology, Dongzhimen Hospital, Beijing University of Chinese Medicine, Beijing, China; ^2^ Department of Cardiology, Dongfang Hospital, Beijing University of Chinese Medicine, Beijing, China; ^3^ Center for Basic Experiments, Beijing Hospital of Traditional Chinese Medicine (Beijing Institute of Traditional Chinese Medicine), Capital Medical University, Beijing, China

**Keywords:** acute myocardial infarction, inflammatory immune mechanisms, nanoparticle-based drug delivery systems, neutrophils, macrophages

## Abstract

Acute myocardial infarction (AMI) remains a major cause of cardiovascular mortality worldwide. The inflammatory immune response after AMI plays a dual role: it facilitates the clearance of necrotic tissue but can also exacerbate injury, significantly affecting patient outcomes. Conventional anti-inflammatory therapies are often limited by systemic toxicity and insufficient targeting, highlighting the need for more refined approaches. This review systematically examines the interplay between AMI's key inflammatory immune mechanisms—including neutrophil N1/N2 phenotypic switching, macrophage M1/M2 polarization, and Treg/Th17 lymphocyte balance—and advancements in nanoparticle-based drug delivery systems (NP-NDDSs) designed to target these mechanisms. NP-NDDSs utilize properties such as size-dependent accumulation, surface functionalization, and stimuli-responsive release (e.g., to pH, ROS, or enzymes) to improve spatiotemporal control over drug delivery. Various nanocarriers, including organic (e.g., liposomes, polymers), inorganic (e.g., gold, silica), and biomimetic (e.g., cell membrane- or exosome-based) systems, have shown potential in influencing neutrophil extracellular trap formation, macrophage phenotype, and lymphocyte activity. These developments suggest that NP-NDDSs could help control excessive inflammation, support tissue repair, and limit adverse remodeling. Nevertheless, challenges in targeting precision, manufacturing scalability, and long-term biosafety remain to be addressed. By summarizing current advances and identifying future needs, this review aims to provide a basis for developing targeted therapies against immune-mediated injury in AMI.

## 1 Introduction

The widespread adoption of coronary revascularization and guideline-directed medical therapies has contributed to a progressive decline in age-standardized mortality after acute myocardial infarction (AMI) (1, 2). Nevertheless, the incidence of recurrent adverse events, such as heart failure-related hospitalizations and death, remains substantial following MI (3–5). After AMI, cardiomyocyte necrosis leads to the release of damage-associated molecular patterns (DAMPs), which initiate an inflammatory cascade. This response has a dual role (6) in the early phase, it supports the clearance of necrotic tissue and promotes repair; however, excessive or prolonged infiltration of neutrophils and monocytes may aggravate myocardial injury (7) and contribute to adverse ventricular remodeling and heart failure (8, 9). Thus, careful modulation of the inflammatory response is considered important for improving outcomes after AMI (10).

Current treatment strategies for MI are limited by the systemic side effects and poor targeting of conventional anti-inflammatory agents. Although drugs such as IL-1β inhibitors, colchicine, and corticosteroids can reduce inflammation, their systemic use is often associated with undesirable effects. Moreover, the complex myocardial microenvironment limits efficient drug delivery, compromising therapeutic efficacy (11, 12).

To address these limitations, nanoparticle-based drug delivery systems (NP-NDDSs) have attracted growing interest. Engineered nanoparticles (NPs) provide a versatile platform due to their tunable physicochemical properties, which can enhance stability and biocompatibility. By optimizing the NP core material, these systems improve drug encapsulation and enable sustained release, reducing premature degradation *in vivo* (13, 14). Additionally, surface modification with targeting ligands (e.g., antibodies or peptides) promotes accumulation within diseased tissues, increasing therapeutic specificity and limiting off-target effects (15).

In cardiovascular applications, NP-NDDSs may help reduce systemic side effects through targeted delivery designs (16) and extend the therapeutic window by controlling drug release kinetics (17). These systems have also been shown to deliver anti-inflammatory agents to neutrophils, curbing excessive inflammation and ameliorating myocardial injury (18, 19). Similarly, NP-NDDSs can modulate macrophage phenotype and function, which may improve cardiac recovery after AMI (19, 20).

Although clinical translation remains challenging—particularly in optimizing targeting efficiency and scaling up manufacturing (21)—continuing advances in NP engineering may help address some limitations of conventional drug delivery. NP-NDDSs represent a promising strategy for precision medicine (22) and could play a role in regulating inflammatory and immune processes in AMI. This review systematically outlines recent advances in NP-NDDSs for managing post-AMI inflammatory injury and supporting myocardial repair, with a focus on targeting strategies, mechanisms of action, and translational challenges. The aim is to offer a reasoned foundation for developing effective and safe NP-based therapies for AMI.

## 2 Pathomechanisms of inflammation and immunity in AMI

Extensive cardiomyocyte death, activation of the innate immune system, and widespread inflammation are common pathological features of acute myocardial infarction (AMI). The acute inflammatory response is a key determinant of final infarct size and the development of adverse ventricular remodeling, highlighting its modulation as an important cardioprotective goal (23). Post-AMI inflammation in the myocardium progresses through three distinct phases: the alarm phase, the leukocyte mobilization phase, and the resolution phase (24). During the alarm phase, dying cardiomyocytes and other cells release signaling molecules known as DAMPs such as high-mobility group box 1 protein (HMGB1) (25), heat shock proteins (HSPs) (26), and fibronectin (27). DAMPs bind to pattern recognition receptors (PRRs) —including Toll-like receptors (TLRs), nucleotide-binding oligomerization domain (NOD)-like receptors, and the receptor for advanced glycation end products (RAGE) —thereby initiating innate immune pathways (28). Key pro-inflammatory signaling pathways activated in innate immune cells include the NOD-like receptor family pyrin domain-containing 3/interleukin-1 beta (NLRP3/IL-1β) pathway (29), the Toll-like receptor 4/nuclear factor kappa-light-chain-enhancer of activated B cells (TLR-4/NF-κB) pathway (30) and the Janus kinase-signal transducer and activator of transcription (JAK-STAT) signaling pathway (31).

**Figure 1:**
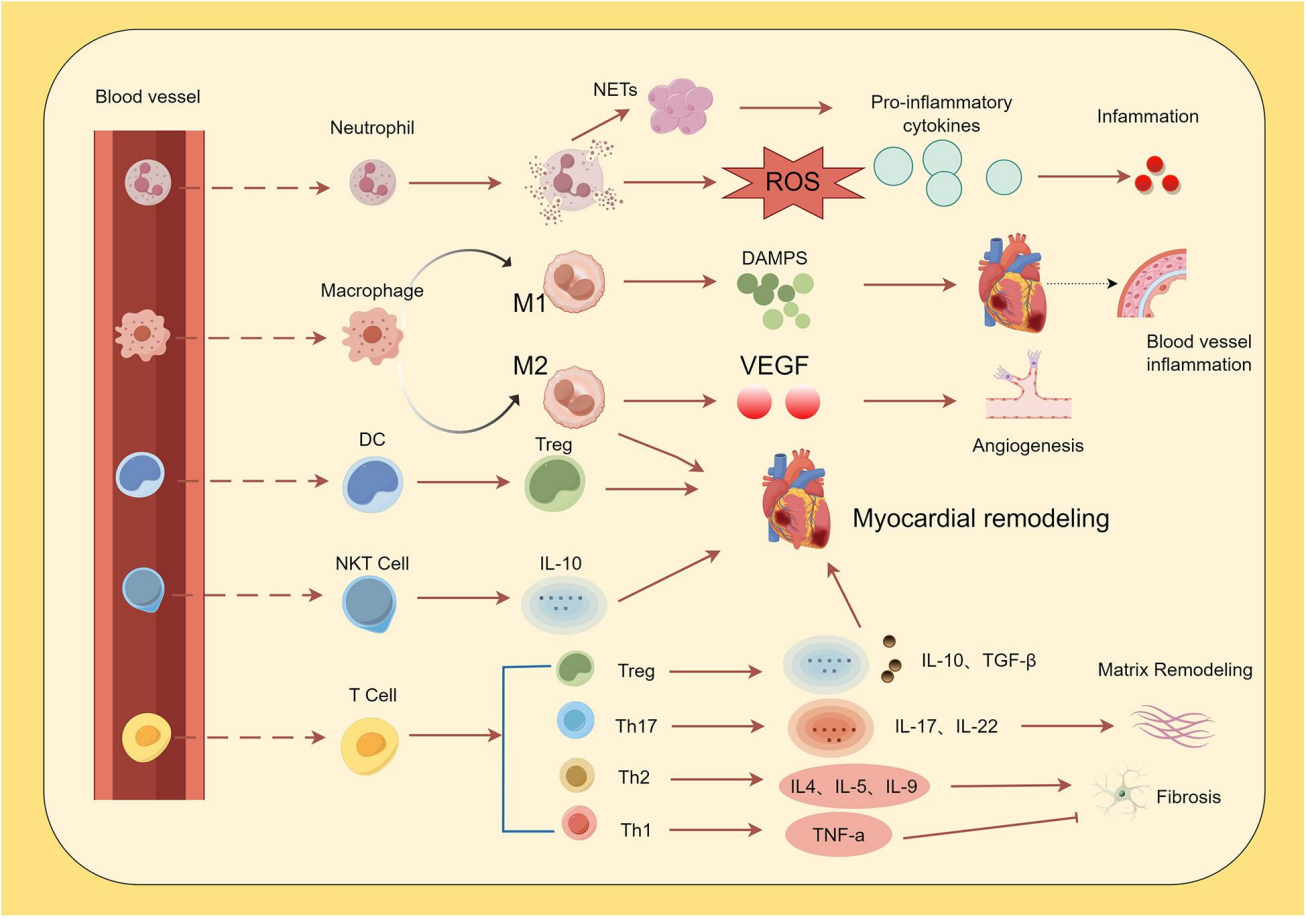
Immune cells in the cardiovascular system. Following acute myocardial infarction (AMI), pro-inflammatory cytokines induce neutrophil infiltration. Neutrophils exacerbate and sustain the inflammatory response by releasing reactive oxygen species (ROS) via neutrophil NETosis and secreting inflammatory cytokines. Monocytes are recruited into cardiac tissue and differentiate into M1 and M2 macrophages. M1 macrophages promote vascular inflammation through the release of damage-associated molecular patterns (DAMPs), while M2 macrophages facilitate repair of damaged tissue via the production of factors such as vascular endothelial growth factor (VEGF). Dendritic cells (DCs) induce myocardial hypertrophy and improve ventricular remodeling by mediating increased regulatory T cell (Treg) activity. Natural killer T (NKT) cells secrete cytokines (e.g., *IL-10*), contributing to the attenuation of inflammation and modulation of ventricular remodeling. Among T cells, Th1 cells reduce fibrotic responses, whereas Th2 and Th17 cells promote fibrosis. Th17 cells additionally drive inflammation and extracellular matrix remodeling, while Treg cells attenuate inflammatory responses.

As summarized in Figure 1, the dynamic interplay among these immune cells—from the initial neutrophil infiltration to the subsequent activation of macrophages, dendritic cells, and lymphocyte subsets—critically shapes the inflammatory landscape and repair processes following AMI. Within the first 30 min to 3 h after AMI, monocytes and neutrophils migrate into the infarct zone under the influence of DAMPs and chemokines (32). Infiltrating monocytes differentiate into macrophages, progressively replacing resident cardiac macrophages (33). However, activation of granulocytes, monocytes, and macrophages—along with the release of pro-inflammatory cytokines, bioactive substances, and neutrophil extracellular traps (NETs)—can also induce additional myocardial damage (34).

In the resolution phase, NETs released by neutrophils activate the NLRP3 inflammasome in macrophages (7). As pro-inflammatory processes subside, macrophages shift toward an anti-inflammatory, pro-resolving M2 phenotype, a transition in which neutrophils play an important role (35). Macrophage function then changes from phagocytosis and extracellular matrix degradation to angiogenesis and granulation tissue formation (36), which may help limit adverse outcomes after AMI (37). Accordingly, a prolonged inflammatory phase can exacerbate myocardial injury, leading to infarct expansion and adverse remodeling (38).

Dendritic cells (DCs), as efficient antigen-presenting cells, help bridge innate and adaptive immunity. Tolerogenic dendritic cells (tDCs), a specific DC subtype, promote systemic activation of regulatory T cells (Tregs) after AMI (39). Activated Tregs participate in modulating myocardial inflammation and facilitate the shift of macrophages from the M1 to the M2 phenotype, thereby supporting favorable ventricular remodeling. Depletion of Tregs impairs resolution-phase functions, resulting in persistent M1 macrophage activity and delayed tissue repair (40).

The adaptive immune system, involving T and B lymphocytes, also contributes to the regulation of post-AMI inflammation. Intramyocardial T-cell recruitment peaks 5–7 days after AMI (41). CD8+ T cells may exacerbate inflammation by promoting cardiomyocyte apoptosis and activating inflammatory macrophages, though they also appear to influence fibrosis and remodeling (42, 43). Among CD4+ T cells, Th1 and Treg subsets are predominant: Th1 cells help maintain a balance between inflammation and repair via secretion of interferon-γ (IFN-γ), interleukin-6 (IL-6), and tumor necrosis factor (TNF) (44), while Tregs exert protective effects by suppressing cardiomyocyte apoptosis and excessive fibrosis (45). Notably, a systemic imbalance between T helper 17 (Th17) and Tregs may exacerbate the inflammatory responses (37).

### 2.1 Role of neutrophils in inflammation-injury and repair

**Figure 2:**
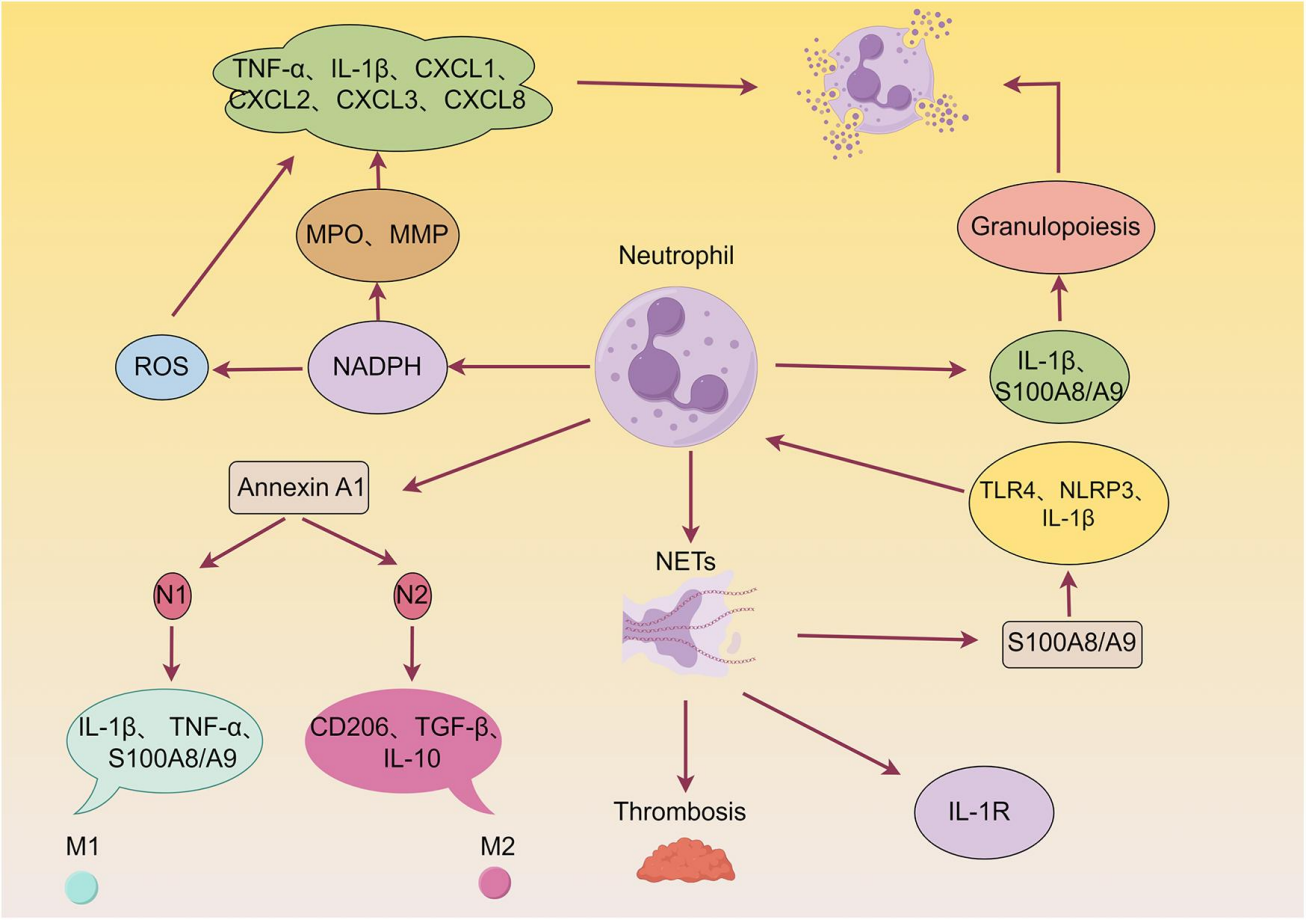
Detrimental and beneficial roles of neutrophils in myocardial infarction (MI) wound healing. Neutrophils are known to exacerbate myocardial injury by releasing reactive oxygen species (ROS), granular components, and pro-inflammatory mediators. Furthermore, neutrophils can form neutrophil extracellular traps (NETs), thereby promoting thrombosis and cardiac damage. Additionally, neutrophils enhance granulopoiesis, establishing a positive feedback loop that amplifies neutrophil production and acute inflammation. Conversely, neutrophils contribute to inflammation resolution, angiogenesis, and scar formation by producing various pro-repair factors, such as Annexin A1 (AnxA1).

Neutrophils are the first innate immune cells to infiltrate ischemic tissue within hours following AMI. The dual role of neutrophils, encompassing both detrimental effects (e.g., ROS release, NETosis) and beneficial contributions, is schematically illustrated in Figure 2. Shortly after AMI onset (within hours), neutrophils rapidly transmigrate across the endothelium via interactions between surface integrins and endothelial adhesion molecules. Their infiltration into the ischemic myocardium is orchestrated by DAMPs and alarmins (46–49). This recruitment is driven by a dual signaling mechanism: necrotic cardiomyocyte-derived DAMPs are sensed by PRRs on resident macrophages and endothelial cells, which in turn release chemokines that establish a gradient guiding neutrophils to the infarct zone (48, 50). Although neutrophils contribute to clearing necrotic debris, they also induce secondary myocardial injury through the release of reactive oxygen species (ROS), proteolytic enzymes, and inflammatory mediators, reflecting their dual role (51).

At the molecular level, neutrophil-derived ROS generated via NADPH oxidase pathways contribute to structural damage by oxidizing cellular components such as proteins and lipids (52, 53), and further amplify local inflammation by promoting pro-inflammatory cytokine secretion (e.g., IL-6, IL-1β) (54). Concurrently, degranulation products-including myeloperoxidase (MPO), elastase, and matrix metalloproteinases (MMPs)-aggravate cardiac remodeling by inducing cardiomyocyte apoptosis, degrading extracellular matrix (ECM), and triggering the release of additional cytokines and chemokines (e.g., TNF-α, IL-1β, CXCL-1-8) (49, 55). These factors may also impair cardiomyocyte contractility through disruption of calcium homeostasis (56), collectively sustaining acute phase injury (7).

Activated neutrophils release NETs, composed of decondensed chromatin and granular proteins, which contribute to AMI pathophysiology through multiple pathways: (1) Promoting microvascular thrombosis (57, 58); (2) Activates the Toll-like receptor 4 (TLR4)/NLRP3/IL-1β signaling axis to stimulate hematopoietic stem/progenitor cells, leading to increased neutrophil production (7, 59); and (3) mediating further neutrophil recruitment via IL-1R, forming a self-amplifying inflammatory circuit that exacerbates myocardial edema and fibrosis (60, 61).

As the pathology evolves (days 1–7 post-AMI), neutrophils undergo phenotypic switching (N1 to N2), which is implicated in repair regulation. In the acute phase (days 1–3), N1 neutrophils promote M1 macrophage polarization via IL-1β and TNF-α secretion. During the repair phase (days 4–7), N2 neutrophils support M2 macrophage polarization through upregulation of CD206, TGF-β, and IL-10 (62, 63). This transition involves Annexin A1, which, upon activation, interacts with formyl peptide receptors (FPRs) to suppress excessive inflammation and promote a pro-angiogenic macrophage phenotype, thereby facilitating tissue repair (64–67). Annexin A1 additionally modulates neutrophil activity through anti-inflammatory, pro-apoptotic, and pro-resolving mechanisms (68, 69).

Notably, S100A8/A9—primarily released from NETs—further modulates inflammatory and reparative processes post-MI. It activates the TLR4/NLRP3/IL-1β signaling axis, stimulating hematopoietic stem/progenitor cells to amplify neutrophil production (7, 59). Moreover, S100A9 exerts a unique time-dependent bidirectional regulatory effect: short-term inhibition (within 3 days) attenuates inflammation and improves outcomes (70), whereas long-term blockade (up to 21 days) impairs the generation and efferocytic function of Ly6CloMerTKhi macrophages via the Nur77 signaling pathway, ultimately worsening cardiac injury (71). This temporal specificity aligns with pathological findings from neutrophil depletion experiments (72), highlighting the need for spatiotemporally precise therapeutic strategies that target neutrophil-mediated responses—such as suppressing excessive acute-phase activation while promoting phenotypic switching during repair—to balance injury containment and tissue regeneration. Thus, targeting S100A8/A9 represents a promising therapeutic approach under active investigation.

### 2.2 Role of monocytes/macrophages in inflammation-injury and repair

**Figure 3:**
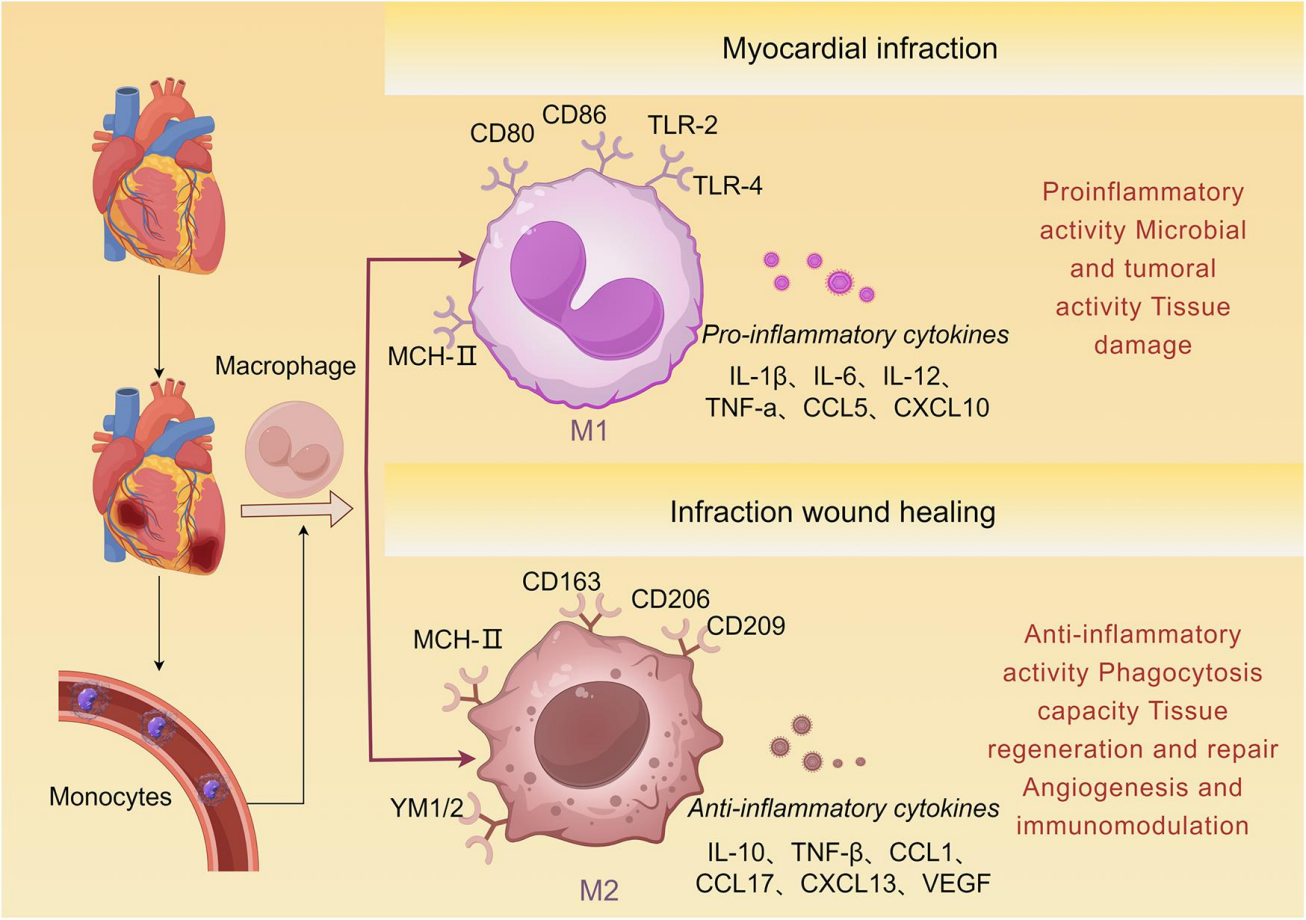
Monocyte differentiation following AMI. Bone marrow-derived monocytes are recruited to the damaged myocardial tissue and subsequently differentiate into M1 and M2 macrophages. M1 macrophages express markers such as TLR-2, TLR-4, CD80, and CD86, and produce pro-inflammatory cytokines including IL-1β, TNF-α, and CCL5. M2 macrophages express markers such as CD163, CD206, and CD209, and produce anti-inflammatory cytokines including IL-10, TGF-β, and VEGF, which induce tissue repair.

The monocyte-macrophage system, part of the myeloid lineage, exhibits spatiotemporal dynamics in composition and function. Monocytes originate from bone marrow and extramedullary hematopoietic sites (e.g., spleen). After maturation, they enter the bloodstream and migrate to peripheral tissues, where they differentiate into macrophages or dendritic cells and participate in immune defense, inflammation regulation, and tissue repair (73). In ischemic heart disease, macrophages play a dual role: they can exacerbate injury through pro-inflammatory responses while also promoting repair via anti-inflammatory mechanisms (74–76), as illustrated in Figure 3 (74–76).

Following AMI, the population of cardiac-resident macrophages declines sharply, while circulating monocytes are extensively recruited to the infarct zone (77). These recruited monocytes originate not only from the spleen but also from extramedullary sources under the regulation of IL-1β (78). Within 24 h post-AMI, classical monocytes preferentially infiltrate the infarct area. Driven by cytokines such as TNF-α and IFN-γ released from injured cardiomyocytes and immune cells, they polarize predominantly toward the classically activated (M1) phenotype in the early phase (78–80). M1 macrophages highly express pro-inflammatory cytokines (e.g., TNF-α, IL-6), cytotoxic mediators (e.g., NO, ROS), and phagocytosis-associated proteins (81). Although they help clear necrotic debris and degrade extracellular matrix, excessive M1 activation may exacerbate the inflammatory microenvironment. In addition, pro-inflammatory exosomes (e.g., containing miR-155) released by M1 macrophages can inhibit angiogenesis, contributing to secondary myocardial injury (19, 82). Upregulation of interleukin-1 receptor-associated kinase-M (IRAK-M) in the infarct microenvironment has been shown to suppress M1 macrophage overactivation, thereby attenuating adverse cardiac remodeling (81).

As the pathology progresses (approximately days 3–7), a phenotypic shift occurs from pro-inflammatory M1 to anti-inflammatory M2 macrophages, which begin to dominate and help protect the heart from adverse outcomes (83). M2 macrophages secrete mediators such as IL-10, CCL17, VEGF, and TGF-β, which stimulate fibroblast activation, extracellular matrix synthesis, and angiogenesis, thereby promoting tissue repair (84, 85). This polarization process is influenced by several mechanisms, including efferocytosis, cell-cell contact signals, anti-inflammatory factors released by neutrophils (e.g., NGAL) (80), and extracellular vesicles carrying anti-inflammatory miRNAs. M2 macrophages specifically express the Mer tyrosine kinase (MerTK) receptor, which enables efficient clearance of necrotic cardiomyocytes through recognition of phosphatidylserine on apoptotic cells. Loss of MerTK disrupts phagocytic function and impedes repair (86). Importantly, the balance between M1 and M2 macrophages is essential for myocardial healing-persistent M1 activation can prolong inflammation, while impaired M2 function may suppress fibrosis resolution and angiogenesis, ultimately worsening cardiac function (87).

Macrophage origin, abundance, and phenotypic heterogeneity further influence the course of post-AMI injury and repair (83). Based on CCR2 expression, cardiac macrophages are classified into two main subsets: CCR2+ macrophages, which are derived from circulating monocytes and recruited to the infarct via the MyD88-dependent pathway. They exhibit M1-like characteristics, express pro-inflammatory cytokines such as CCL2, drive sustained monocyte infiltration, and promote adverse remodeling (88, 89). In contrast, CCR2−macrophages are primarily cardiac-resident cells of embryonic origin, maintained by self-proliferation. They help suppress monocyte recruitment (90), support coronary development, and facilitate cardiac regeneration (91).

Different macrophage subpopulations cooperate during cardiac injury and repair. Recent studies reveal that small extracellular vesicles derived from M2 macrophages can reduce CCR2^+^ macrophage abundance, limit monocyte recruitment to the infarct, and promote M1-to-M2 phenotypic conversion, thereby enhancing angiogenesis and improving myocardial repair (81). These findings suggest that targeting of macrophage function and phenotypic switching may hold potential for reducing adverse remodeling and supporting cardiac repair after AMI (92).

### 2.3 Role of lymphocytes and dendritic cells in inflammation-injury and repair

T cells play a dual role in the post-AMI inflammatory response, involving a dynamic balance between pro- and anti-inflammatory mechanisms. During ischemia-reperfusion injury, CD4^+^ T cells-particularly the Th1 subset-can influence infarct size by releasing IFN-γ and IL-17, cytokines associated with cardiomyocyte death and fibroblast proliferation (93, 94). CD8^+^ T cells show a more complex role: although impaired CD8^+^ T cell function has been linked to better initial cardiac recovery, their absence delays necrotic tissue clearance, impairing scar formation and increasing the risk of cardiac rupture (95).

Regulatory T cells (Tregs) generally support cardiac repair (45, 96). They help modulate the post-AMI immune environment by suppressing CD8^+^ T cell activity (97) and influencing monocyte/macrophage differentiation (96). Studies in animal models indicate that increasing Treg numbers through exogenous administration improves cardiomyocyte survival, cardiac function, and repair outcomes, partly by reducing pro-inflammatory monocytes/macrophages and encouraging a reparative macrophage phenotype (98).

Dendritic cells (DCs), as key antigen-presenting cells, also contribute to immune regulation and repair after AMI. Tolerogenic DCs (tDCs), a specific subset, help activate Tregs and modulate macrophage polarization. In DC-depleted mice, post-infarction ventricular remodeling is more severe, with increased inflammatory monocytes, macrophages, and cytokines, as well as higher rupture risk, supporting a protective role for DCs (40, 99, 100). By promoting Treg activation, tDCs facilitate a shift from M1 to M2 macrophages, thereby improving cardiacfunction (101). In mouse models, tDC administration after AMI reduces infarct size, improves systolic function, and enhances survival. Imaging and molecular analyses show that tDC treatment promotes Treg infiltration, elevates anti-inflammatory cytokines (e.g., IL-4, IL-10) and VEGF, and accelerates the M1-to-M2 transition, contributing to reduced inflammation and better tissue repair (40, 102, 103). Overall, tDCs help attenuate inflammation, support tissue repair, and improve cardiac outcomes after AMI through Treg activation, macrophage polarization, and stimulation of angiogenesis. These findings indicate that targeting DC subsets may offer therapeutic potential in myocardial infarction and heart failure.

B lymphocytes contribute to immune responses through antibody production, antigen presentation, and cytokine secretion. Certain B cell subsets expand in pericardial adipose tissue and accumulate in the infarcted heart after AMI, where they may exert anti-inflammatory effects via IL-10, potentially helping to resolve inflammation (104). In a clinical study of 14 MI patients, higher B cell levels after PCI correlated with improved LVEF. Mouse studies further showed that empagliflozin enhanced cardiac repair by restoring naïve B cell number and function, and infusion of B cells improved cardiac function and reduced infarct size, supporting a protective role (105). However, the exact mechanisms by which B cells regulate post-AMI immunity require further investigation.

## 3 Therapeutic advantages of nanoparticle-based drug delivery in AMI

A central challenge in current cardiovascular disease therapy is the targeted delivery of therapeutics to specific pathological sites—such as areas of inflammation, thrombosis, or abnormal cell proliferation—while minimizing effects on healthy tissues.

**Figure 4:**
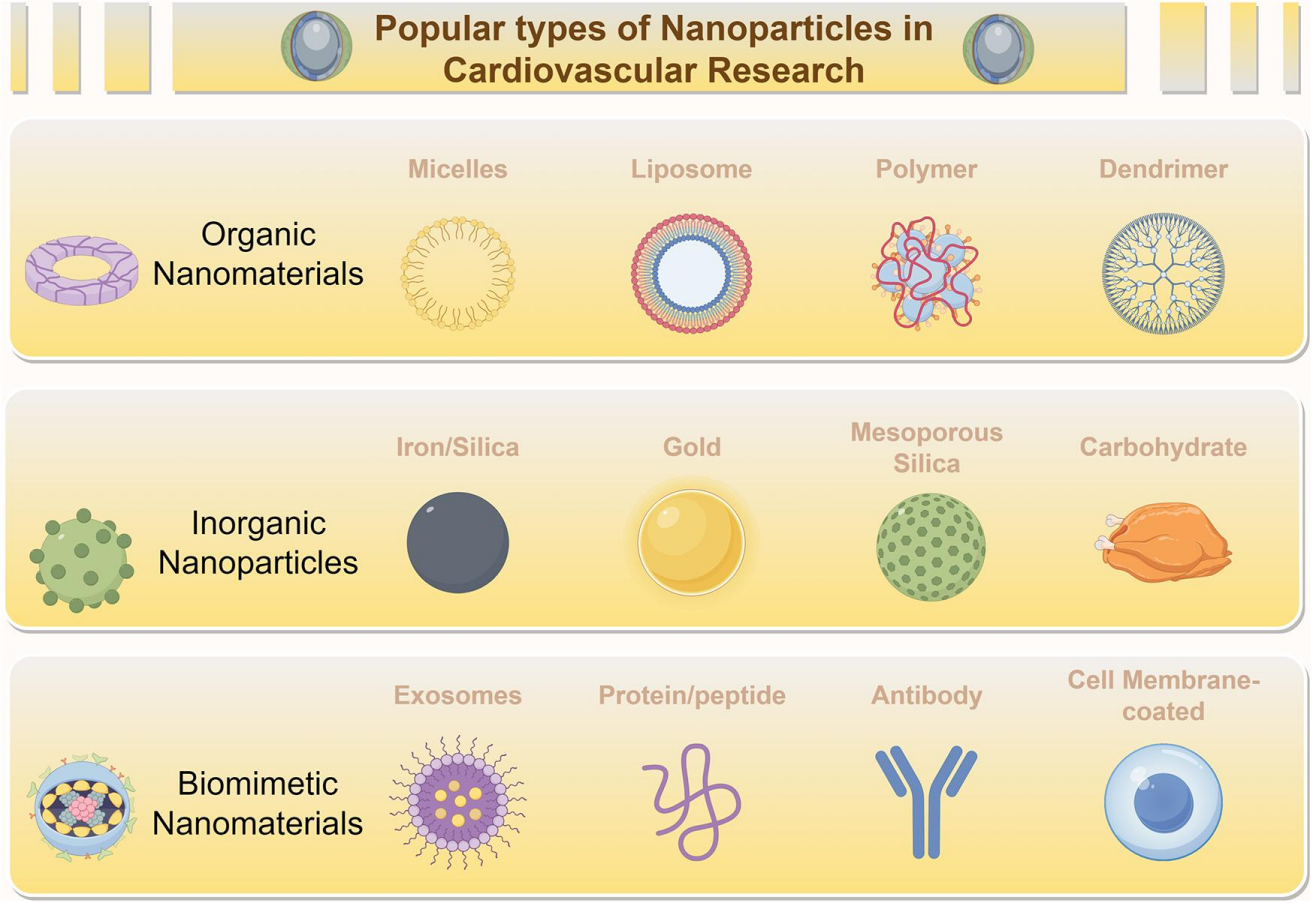
Diverse nanoparticles (NPs) employed for treating cardiovascular diseases. NPs, including Organic Nanomaterials (e.g., Micelles, Liposomes, Polymers, Dendrimers), Inorganic Nanoparticles (e.g., Iron/Silica, Gold, Mesoporous Silica, Carbohydrate-based NPs), and Biomimetic Nanomaterials (e.g., Exosomes, Protein/peptide-based NPs, Antibody-conjugated NPs, Cell Membrane-coated NPs), are widely employed in the treatment of myocardial ischemic diseases.

In recent years, nanoscale materials, particularly NPs, with dimensions on the nanometer scale, have become important tools in modern medicine. Among these, the types most commonly used in the field of cardiovascular medicine are shown in Figure 4. Their applications range from targeted gene delivery to contrast enhancement in medical imaging (106, 107). NPs utilize their distinct size, tunable physicochemical properties, and chemical composition to facilitate transport through tissues and the bloodstream for drug delivery. They also exhibit increased chemical reactivity, energy absorption, and biodistribution capabilities (108). This rapidly evolving field provides new therapeutic opportunities for a variety of diseases (109) (Figure 4).

The medical utility of NPs is mainly reflected in three areas: extending the drug half-life and reducing systemic toxicity to optimize the therapeutic window; enhancing targeting specificity by modifying physicochemical properties such as surface charge to limit off-target effects; and improving drug accumulation at disease sites through combined active and passive targeting strategies (110). In the treatment of coronary heart disease, delivery systems based on biodegradable organic carriers—such as liposomes, micelles, and polymeric NPs, often functionalized with targeting ligands (e.g., antibodies, peptides) or functional polymers—have shown improved therapeutic efficacy (111–113).

NPs facilitate drug delivery through several advantageous mechanisms: sustaining drug release to prolong *in vivo* residence time; providing controlled release kinetics that help reduce side effects such as abnormal vascular growth or vascular leakage; and enabling precise delivery to specific sites such as ischemic regions, thereby improving treatment outcomes (114). This targeting ability partly arises from the capacity of NPs to exploit pathological microenvironment features—such as ischemia-induced vascular permeability—for enhanced accumulation in target tissues like the ischemic myocardium (115). Moreover, the inherent stimulus-responsiveness of NPs (to temperature, pH, or external stimuli such as ultrasound) allows spatiotemporally controlled drug release (116, 117).

### 3.1 Advanced NP-NDDSs for myocardial infarction therapy

**Figure 5:**
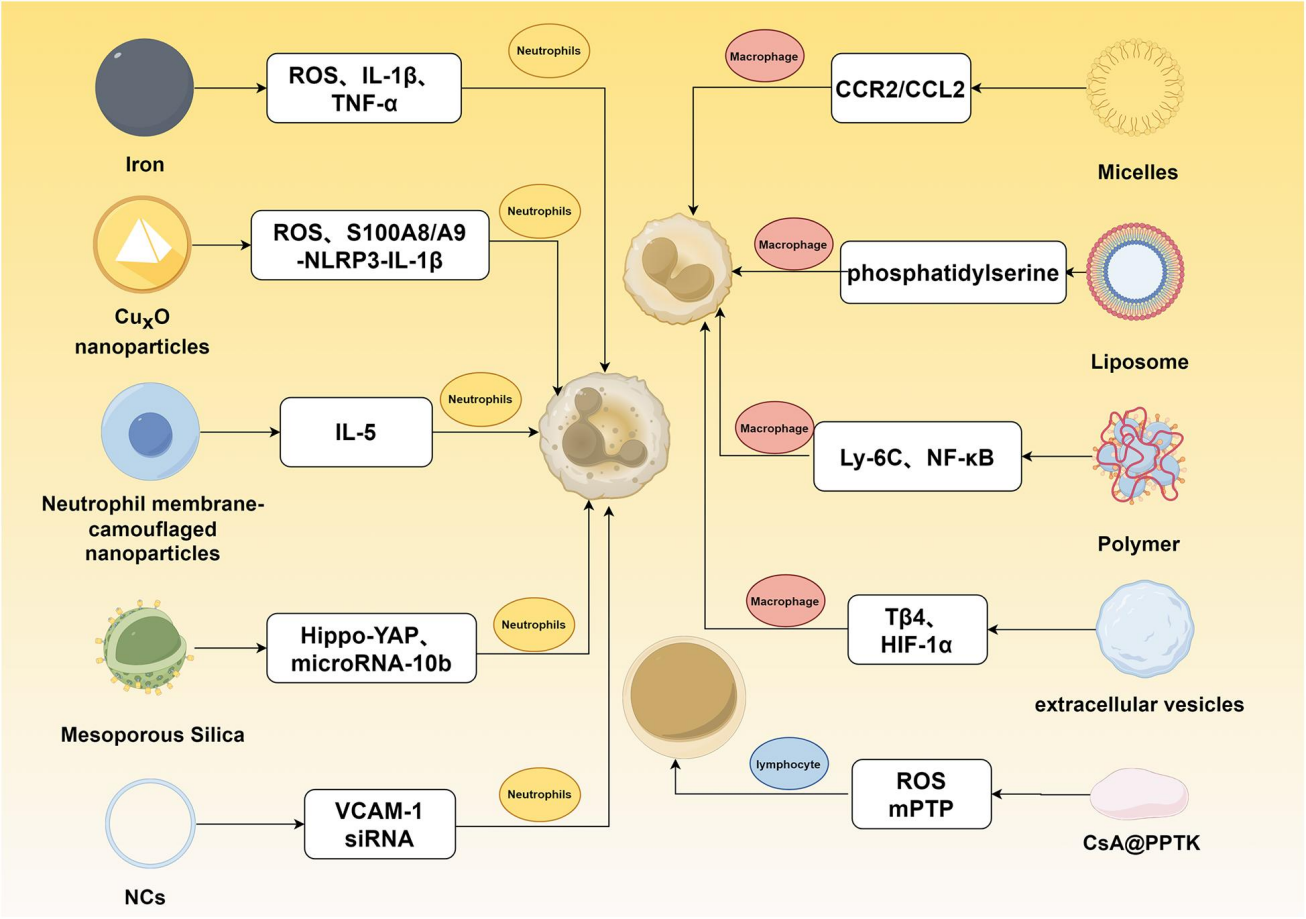
Mechanisms of nanomaterial-mediated immunomodulation for myocardial infarction intervention. This figure illustrates the targeting of distinct pathways in neutrophils, macrophages, and lymphocytes for treating myocardial infarction.

A variety of nanoparticle (NP) classes, including organic systems such as liposomes, micelles, dendrimers, and polymeric NPs, as well as inorganic carriers like gold and silica NPs, provide various approaches for targeted drug and gene delivery to the heart. To visually organize the diverse array of NPs discussed in this section, Figure 5 provides a schematic overview of the major NP classes (organic, inorganic, biomimetic) and their immunomodulatory mechanisms targeting different immune cells in MI. Emerging biomimetic nanoplatforms are further expanding possibilities in this area.

Organic NPs, which include liposomes, micelles, and dendrimers, generally exhibit tunable biocompatibility, reduced toxicity, and improved drug delivery efficiency through adjustment of their chemical and structural properties (118). Their nanoscale size (1–100 nm) allows size-dependent effects and customizable surface chemistry, enabling advances in gene delivery, medical imaging, and targeted drug transport (106, 119).

Research on organic NP-based drug delivery systems has largely centered on liposomes, micelles, polymeric NPs, and dendrimers (113). Micelles are colloidal particles formed by the self-assembly of amphiphilic molecules, with a hydrophobic core that encapsulates poorly soluble drugs and a hydrophilic shell that improves solubility. Their small size (typically under 80 nm) promotes better penetration into ischemic myocardial tissue compared to larger NPs (120). By incorporating targeting groups such as antibodies or ROS-sensitive peptides, micelles can be engineered to recognize specific components in atherosclerotic plaques, improving targeting accuracy (121). Preclinical studies suggest that targeted micelles carrying anti-inflammatory or anti-angiogenic drugs can extend circulation half-life, lower pro-inflammatory cytokine levels, and reduce plaque area (120, 122).

Polymers are widely used as NP materials due to their low toxicity and versatility for chemical modification (123). Both natural polymers (e.g., starch, cellulose) and synthetic ones such as PLA, PGA, and PLGA have been utilized (124). PLGA, in particular, is known for its controllable degradation and is often used for sustained drug release. For example, PLGA NPs prepared by emulsion solvent diffusion have been used to deliver glutathione or heparin to the heart within 2 h in models of myocardial ischemia-reperfusion injury (MIRI) (125, 126).

Dendrimers are highly branched, well-defined macromolecules whose size can be controlled by the number of synthetic generations. Their multifunctional surfaces allow efficient loading of drugs, imaging agents, and targeting molecules such as folic acid or antibodies (127). With optimized conjugation methods, dendrimers represent potentially useful platforms for cardiovascular disease diagnosis and targeted treatment (128).

Liposomes are self-assembled phospholipid bilayer vesicles that mimic cell membranes and can carry both hydrophilic and hydrophobic drugs. They are especially useful for co-delivering genes and drugs, and they offer prospects for scalable production (129). However, issues such as drug leakage and particle aggregation may affect release kinetics, indicating a need for further formulation improvement (130).

Inorganic nanocarriers, including silica-, carbon-, and metal-based NPs (e.g., AuNPs), often show high physicochemical stability and strong drug-loading capacity, supporting precise delivery (131, 132). Gold NPs (AuNPs) are easily synthesized, exhibit low toxicity and minimal immunogenicity, and have been used to deliver cardioprotective drugs such as Simdax. In heart failure models, AuNP-conjugated Simdax showed better efficacy than the free drug, likely due to improved tissue targeting (133). Silica NPs provide high surface area and a mesoporous structure that can be functionalized for efficient drug or gene delivery, as seen in adenosine delivery to MIRI-affected heart tissue (134). However, the relatively low biodegradability of inorganic NPs compared to organic ones has raised concerns about long-term accumulation and potential toxicity. Complicated surface modification processes also present challenges, highlighting the importance of developing controlled degradation and functionalization methods to improve biosafety and efficacy (131, 135).

Although traditional nanocarriers are widely used, emerging biomimetic nano-delivery systems combine nanotechnology with biomimetic principles to provide alternative strategies for treating AMI and MIRI (136–138). These systems employ nanoscale carriers (1–1,000 nm) designed to imitate biological structures. Examples include cell membrane-coated nanoparticles, nano-sized extracellular vesicles such as exosomes, and nanozymes—nanomaterials that mimic the catalytic activity of natural enzymes.

Biomimetic systems allow efficient encapsulation and targeted delivery of therapeutics while leveraging natural biological mechanisms to avoid immune clearance, extend circulation time, and increase accumulation in ischemic heart tissue (139, 140) For instance, nanoparticles coated with autologous cell membranes or exosome-like vesicles inherit the targeting ability and biocompatibility of the source cells, functioning similarly to liposomes but with enhanced bio-specificity (141). Biomimetic nanozymes can reduce oxidative stress by scavenging excess ROS (142, 143). In comparison with conventional nanocarriers, biomimetic systems often improve the solubility and stability of poorly soluble drugs and allow stimuli-responsive release in response to microenvironmental signals such as pH changes or enzyme activity in ischemic regions. This can help minimize off-target effects and related side effects (144–146). One example is a neutrophil membrane-camouflaged delivery system loaded with siRNA. Modified with integrins to improve targeting and hemagglutinin to promote endosomal escape, this system enhanced siRNA delivery in MIRI models, reduced neutrophil infiltration and microthrombus formation, limited infarct size, and improved cardiac function (147).

### 3.2 Neutrophil-targeting nanoparticle-based drug delivery systems

Emerging evidence indicates that the initial response to myocardial tissue damage triggers intense neutrophil-dominated inflammation, exacerbating injury and potentiating ventricular remodeling. Recent advances leverage nanomedicine to precisely modulate neutrophil functions, offering cardioprotective effects through extended drug efficacy and enhanced targeting.

#### 3.2.1 Therapeutic targeting of S100a8/A9 in neutrophil-driven inflammation

As primary early responders in inflammation, neutrophils secrete the alarmin S100A8/A9, which promotes the infiltration of innate immune cells such as macrophages and drives their polarization toward the pro-inflammatory M1 phenotype. Therefore, inhibiting the S100A8/A9 signaling axis has emerged as a common therapeutic strategy. Beyond multi-enzyme-mimetic nanocatalysts, multifunctional nanocomplexes have been developed to produce synergistic effects. For example, dual-function nanocomplexes combining CuxO nanoparticles—which quench ROS through multi-enzyme mimicry—and the S100A8/A9 inhibitor ABR-25757 can synergistically block the S100A8/A9-NLRP3-IL-1β pathway, contributing to a reduction in infarct size (148). Further advances include targeted pathway inhibition and gene silencing strategies. Based on the concept of S100A8/A9 pathway blockade, receptor-mediated siRNA delivery systems have been designed to disrupt neutrophil-driven inflammation with greater precision. One such system uses S100A9-siRNA nanoparticles coated with engineered macrophage membranes functionalized with RAGE (receptor for advanced glycation end products) and hemagglutinin (HA). These particles achieve dual targeting: RAGE facilitates binding to S100A9, which is highly expressed in the infarcted myocardium, while HA promotes endosomal escape for efficient cytosolic siRNA release. This approach leads to S100A9 gene silencing, inhibition of the S100A8/A9-TLR4 axis, and a subsequent decrease in neutrophil recruitment (20).

#### 3.2.2 Neutrophil membrane biomimetic nanocarriers for modulating the immune microenvironment

Neutrophil membrane-biomimetic nanocarriers, which leverage inherent long circulation and inflammatory homing capabilities, have shown promise as tools for regulating the immune microenvironment after AMI.

(1) Delivering Immunomodulatory Factors: In one study, neutrophil membrane-camouflaged NPs with a PLGA core were loaded with IL-5. After reaching inflammatory sites, these carriers reduced neutrophil infiltration while promoting eosinophil recruitment and M2 macrophage accumulation. They also enhanced angiogenesis through increased AKT and ERK1/2 phosphorylation, leading to improved ventricular remodeling (18).
(2) Adsorbing Pro-inflammatory Mediators: Biomimetic platforms such as neutrophil membrane-cloaked liposomal NPs (Neu-LPs) can adsorb pro-inflammatory cytokines (TNF-α, IL-1β, IL-6) and CXCL-2 in the infarcted heart. This action dampens neutrophil infiltration and accelerates M2 macrophage polarization within 3 days post-AMI, which helps attenuate apoptosis and fibrosis (149).
(3) Co-delivering Therapeutic Nucleic Acids: More advanced systems combine cytokine neutralization with regenerative therapy. For instance, silica NPs cloaked with neutrophil membranes and loaded with miR-10b (NM@miR) can deliver this microRNA to cardiomyocytes, where it suppresses the Hippo pathway to promote proliferation and regeneration (141).

#### 3.2.3 Synergistic delivery and multifunctional nanoplatforms

NPs can be designed to achieve multifunctional immunomodulation through synergistic delivery strategies.

(1) Drug-siRNA Synergistic Delivery: Mengying Hou et al. developed an endothelial cell-targeted, ROS-sensitive nanocomplex (NCs). Its core contains PLGA NPs loaded with the anti-inflammatory drug dexamethasone (DXM). The surface is electrostatically coated with cRGD-PEG-bis(diselenide)-crosslinked polyethylenimine (RPPT) complexed with VCAM-1 siRNA (siVCAM-1). In a MIRI model, cRGD mediates targeting to inflamed endothelium. Local high ROS levels trigger degradation of RPPT, leading to release of siVCAM-1 and silencing of VCAM-1 expression. This effect works together with DXM to inhibit neutrophil chemotaxis and adhesion, thereby alleviating myocardial inflammation and improving cardiac function. This design addresses challenges related to siRNA encapsulation, release, and efficiency-toxicity balance (150).
(2) Membrane Camouflage with Functional Modification for siRNA Delivery: Yaohui Jiang et al. constructed an engineered neutrophil membrane-camouflaged nanodelivery system (MNM/siRNA NPs) modified with integrins and hyaluronic acid (HA) and loaded with siRNA targeting integrin α9. HA facilitates endosomal escape, while integrin α9/β1 enables targeting via binding to endothelial VCAM-1. In a MIRI model, silencing integrin α9 reduced neutrophil infiltration, NET formation, and microthrombosis, resulting in smaller infarct size and improved cardiac function (147).

#### 3.2.4 Mimicking natural biomolecules to regulate leukocyte function

Another strategy involves mimicking natural biomolecules to regulate leukocyte function. Researchers have developed NPs (n-apo AI) composed of human apolipoprotein AI (Apo AI) complexed with soybean phosphatidylcholine, which mimic the structure of high-density lipoprotein (HDL). When administered intravenously after reperfusion, n-apo AI targets neutrophils, reduces surface expression of the integrin CD11b, decreases leukocyte infiltration into the infarct zone, and promotes monocyte polarization toward the anti-inflammatory Ly6Clow phenotype. This supports inflammation resolution and tissue repair. Notably, this approach also showed potential in reducing circulating leukocyte activity in patients with type 2 diabetes, suggesting a possible new direction for managing inflammation after AMI (151) (Table 1).

**Table 1:** Neutrophil-targeting nanoparticle-based drug delivery systems.

Reference	Nanoparticle type	Targeting mechanism	Efficacy endpoints	Therapeutic agent	Administration route	Administration time	Year
(148)	CuxO NPs	Clears ROS and inhibits S100A8/A9; blocks S100A8/A9-NLRP3-IL-1β pathway	Reduced infarct size by 27%, increased LVEF by 22%	S100A8/A9 inhibitor (ABR-2575)	Intravenous	Immediately after AMI	2024
(20)	Engineered Macrophage Membrane-Coated S100A9-siRNA NPs	Targets S100A9 via RAGE binding, silences S100A9 gene to inhibit TLR4 axis	Reduced infarct size by 29%, increased LVEF by 30%	S100A9-siRNA	Intravenous	1 h after reperfusion	2024
(18)	Neutrophil Membrane-Camouflaged PLGA NPs (NM-NPIL-5)	Carries IL-5; reduces neutrophil influx, recruits eosinophils/M2 macrophages, enhances angiogenesis.	Reduced infarct size by 9%, increased LVEF by 23%	IL-5	Intravenous	20 min after reperfusion	2023
(149)	PLGA Nanocomplexes (ROS-sensitive)	Delivers siVCAM-1 via ROS-triggered release; silences VCAM-1 and blocks neutrophil adhesion.	Increased LVEF by 25.7%, decreased neutrophil infiltration by 23%	VCAM-1 siRNA + Dexamethasone	Intravenous	10 min after reperfusion	2022
(141)	Neutrophil Membrane-Coated Silica NPs (NM@miR)	Delivers miR-10b to suppress Hippo pathway, promoting cardiomyocyte proliferation and regeneration.	Increased LVEF by 11%	microRNA-10b	Intravenous	24 h after reperfusion	2023
(150)	Neutrophil Membrane-Cloaked Liposomal NPs (Neu-LPs)	Adsorbs pro-inflammatory cytokines; reduces neutrophil infiltration, accelerates M2 macrophage polarization.	Reduced infarct size by 37%, increased LVEF by 22%	–	Intravenous	1 day after reperfusion	2022
(147)	Integrin *α*9/β1-targeted siRNA NPs (HA-NM camouflaged)	Silences integrin *α*9 to suppress neutrophil infiltration and NETosis.	Reduced infarct size by 37%, increased LVEF by 30%	Integrin α9-targeted siRNA	Intravenous	Immediately after surgery, for 3 days	2025
(151)	Apolipoprotein AI-based NPs (n-apo AI)	Mimics HDL; downregulates CD11b, reduces leukocyte infiltration, promotes anti-inflammatory monocyte polarization.	Elevated the Ly6C^Low^/Ly6C^High^ ratio by approximately 75%	n-apo AI (CSL111)	Intravenous	Immediately after reperfusion	2020

### 3.3 Macrophage-targeting nanoparticle-based drug delivery systems

#### 3.3.1 Targeting monocyte/macrophage recruitment and initial inflammation

Following AMI or MIRI, damaged cardiomyocytes release DAMPs. The recognition of DAMPs by TLR4 on innate immune cells initiates a robust inflammatory response, leading to the recruitment of neutrophils and monocytes/macrophages to the heart. To target this initial trigger, PLGA NPs delivering the TLR4 inhibitor TAK-242 were shown to suppress monocyte/macrophage TLR4/NF-κB signaling, which reduced the infiltration of Ly6Chigh monocytes and the release of pro-inflammatory cytokines such as IL-6 and CCL-2, thereby attenuating acute myocardial inflammation (152).

After AMI, monocytes are recruited to the infarct area mainly through CCR2 binding to its ligand CCL-2. In one approach, anti-CCR2 antibody-modified PEG-DSPE micelles (21–35 nm) were used to deliver a CCR2 antagonist specifically to monocytes. This intervention blocked CCR2/CCL-2 signaling, significantly reduced the migration of spleen-derived monocytes to the heart, and resulted in smaller infarct size and improved cardiac function (153).

The *in vivo* delivery efficiency of NPs is often limited by clearance from the mononuclear phagocyte system. Qiang Long et al. proposed an innovative strategy by targeting the spleen as a key organ for regulating myocardial inflammation. Their work showed that monocytes recruited during acute myocardial reperfusion injury originate from the spleen, and that early expression of Interferon Regulatory Factor 7 (IRF7) in the spleen influences cardiac macrophage function. By developing spleen-targeting biomimetic NPs (RP182-STEER) loaded with HS38, they inhibited early IRF7 expression specifically in the spleen. This approach blocked the egress of pro-inflammatory monocytes to the heart without interfering with IRF7 function during the repair phase, leading to improved acute and chronic outcomes (154).

#### 3.3.2 Modulating macrophage polarization state

The inflammatory outcome after injury is strongly influenced by macrophage phenotype. Promoting a shift from pro-inflammatory (M1) to a reparative (M2) state is considered important for resolution of inflammation and tissue repair. Peroxisome proliferator-activated receptor-γ (PPARγ), a nuclear receptor, can inhibit NF-κB expression in macrophages and encourage transition to an M2 phenotype. In one study, PLGA NPs delivering the PPARγ agonist pioglitazone promoted M2 polarization, which was associated with reduced inflammation and fibrosis, and improved cardiac function after MIRI (146).

Macrophage polarization can also be influenced by directly targeting intracellular signaling pathways. For example, Laura Tesoro et al. developed lipid membrane NPs (NL10) functionalized with an IL-10 receptor-targeting peptide (IT9302) via PEGylated phospholipids. These NIL10 NPs promoted STAT3 activation and inhibited NF-κB nuclear translocation in macrophages, accelerating their shift toward an anti-inflammatory phenotype. This shift was accompanied by increased expression of anti-inflammatory cytokines including IL-4, IL-10, and IL-13, which ultimately contributed to reduced fibrosis and improved cardiac function (155).

Another innovative strategy involves clearing stress-induced senescent cells (SISCs) that arise after injury. Researchers used biodegradable PLGA NPs loaded with the senolytic drug ABT263 (ABT263-PLGA), administered via local injection. These NPs facilitated the clearance of SISCs through macrophage phagocytosis, which led to reduced levels of inflammatory mediators and fibrosis, promoted M2 polarization, and supported functional recovery, while avoiding systemic toxicity (156).

#### 3.3.3 Biomimetic nanocarriers for enhanced targeting and delivery

Biomimetic nanodrug delivery systems are considered promising for myocardial injury therapy due to their biocompatibility and targeting capabilities. For instance, platelet membrane-coated NPs (CsA@PPTK) have been used to deliver Cyclosporine A (CsA) to ischemic myocardium. This approach inhibited the mitochondrial permeability transition pore (mPTP), scavenged ROS, promoted M2 macrophage and Tregs, and provided long-term functional benefits through combined antioxidant, anti-inflammatory, and anti-apoptotic effects (157).

Apoptotic cell membranes also represent useful biomimetic materials. Lili Bao et al. developed neutrophil apoptotic body membrane-coated mesoporous silica NPs loaded with hexyl-5-aminolevulinate hydrochloride (HAL). By mimicking natural apoptosis, this system utilizes adhesion molecules on the apoptotic membrane to target inflammatory sites for specific uptake by macrophages. An esterase-responsive polymer cap then opens, releasing HAL to initiate the heme metabolism pathway and generate bilirubin, an anti-inflammatory metabolite. This process can enhance macrophage polarization toward an anti-inflammatory phenotype, supporting inflammation resolution and tissue regeneration (158). Extracellular vesicles (EVs) also serve as promising natural drug carriers. One example is monocyte membrane-modified extracellular vesicles (Tβ4-MmEVs), which leverage CCR2/CCL-2 targeting and CD47-mediated evasion of clearance to deliver thymosin β4 (Tβ4) and promote angiogenesis and repair (159).

#### 3.3.4 Synergistic strategies: targeting cellular injury, oxidative stress, and inflammation

In addition to modulating cellular migration, synergistic interventions that address early cellular injury (e.g., mitochondrial dysfunction) and inflammation have shown promise. PLGA NPs co-delivering the mPTP inhibitor CsA (targeting CypD) and the CCR2 antagonist pitavastatin were shown to attenuate mitochondrial damage—thereby inhibiting NLRP3 inflammasome activation—and reduce monocyte-driven inflammation, resulting in cardioprotective effects (160).

Excessive ROS production and subsequent oxidative stress play a key role in exacerbating MIRI. Advanced bimetallic nanozyme strategies, such as Cu-TCPP-Mn, incorporate manganese (Mn) and copper (Cu) within Tetrakis(4-carboxyphenyl)porphyrin (TCPP) ligands to form a metal-organic framework (MOF). This nanozyme mimics the cascade activity of superoxide dismutase (SOD) and catalase (CAT) to scavenge ROS. In MI and MIRI models, intravenously administered Cu-TCPP-Mn (20 nm) accumulated in ischemic myocardium, suppressed IL-1β and TNF-α expression, reduced neutrophil and macrophage infiltration, and increased anti-inflammatory IL-10 levels (161). Similarly, a polyglucose-sorbitol carboxymethylether (PSC)-coated Prussian blue nanozyme (PBNz@PSC) exhibited biocompatibility and targeted damaged myocardium. PBNz@PSC scavenged ROS via SOD/CAT-like activity, promoted M2 macrophage polarization, modulated AMPK/NF-κB signaling, enhanced vasodilation, and improved cardiac function (142). In a combined antioxidant and gene therapy approach, cationic cerium dioxide (CeO_2_) NPs delivered an Nrf2 plasmid to macrophages. Using monocyte transport for infarct accumulation, this system activated the Nrf2/ARE antioxidant pathway and suppressed inflammation, leading to functional improvement and reduced damage (162).

#### 3.3.5 Nanocarrier delivery of herbal-derived monomers

Targeted delivery strategies continue to improve treatment precision. For example, CD11b antibody-modified mesoporous silica NPs (MSN-NGR1-CD11b) were used to deliver the traditional Chinese medicine monomer notoginsenoside R1 (NGR1) to CD11b^+^ leukocytes in the infarct zone. This method was associated with improved cardiac function, promoted angiogenesis, reduced apoptosis, and polarized macrophages toward the M2 phenotype (163). In another study, platelet membrane-coated PLGA NPs (BBR@PLGA@PLT NPs) delivered berberine (BBR) to the infarcted myocardium. The platelet membrane improved targeting specificity and reduced liver uptake. This system increased the number of reparative macrophages, decreased inflammatory macrophages and apoptotic cells, and improved cardiac function while reducing fibrosis and promoting angiogenesis, with reported good biosafety (164) (Table 2).

**Table 2:** Macrophage-targeting nanoparticle-based drug delivery systems.

Reference	Nanoparticle type	Targeting mechanism	Efficacy endpoints	Therapeutic agent	Administration route	Administration time	Year
(152)	Polymeric NPs	Inhibits recruitment of Ly-6Chigh monocytes to the heart; suppresses NF-*κ*B activation.	Reduced infarct size by 21%, increased LVEF by 11%	TAK-242 (TLR4 inhibitor)	Intravenous	Immediately after reperfusion	2019
(153)	Micelle-type NPs	Binds CCR2-expressing Ly-6Chigh monocytes post-AMI; blocks CCR2/CCL2 axis to inhibit monocyte recruitment and reduce inflammatory cell infiltration.	Reduced Ly-6C^high^ monocytes by 7%, reduced infarct size by 12.5%, increased LVEF by 6.9%	CCR2 antagonist	Intravenous	48 h & 72 h after MI	2018
(154)	Erythrocyte Membrane-Biomimetic NPs	Inhibits early IRF7 expression in spleen; blocks pro-inflammatory monocyte migration to the heart.	Reduced infarct size by 7.9%, increased LVEF by 8.2%	HS38 (DAPK1 inhibitor)	Intravenous	1 h after reperfusion	2025
(146)	Poly(lactic-co-glycolic acid) NPs	Inhibits Ly6Chigh monocyte recruitment; polarizes macrophages to reparative M2 phenotype to attenuate cardiac remodeling.	Reduced infarct size by 12%, increased LVEF by 10%	Pioglitazone (PPAR*γ* agonist)	Intravenous	Immediately after reperfusion	2019
(155)	Micelle-type NPs	Promotes STAT3 activation and inhibits NF-ĸB nuclear translocation to accelerate macrophage polarization.	Reduced infarct size by 28.52%, increased LVEF by 8%	IT9302 peptide (IL-10 agonist)	Intravenous	24 h after ischemia	2022
(156)	PLGA NPs	Induces macrophage phagocytosis of senescent cells (SISCs); inhibits pro-inflammatory cytokine release; promotes M1→M2 polarization to accelerate tissue repair.	Reduced infarct size by 27%, increased LVEF by 30%	ABT263 (Senolytic)	Intramyocardial	5 min before reperfusion	2021
(157)	Tregs Biomimetic NPs (CsA@PPTK)	Scavenges ROS; inhibits mPTP over-opening; modulates macrophage phenotype (↑M2, ↓M1) and promotes Treg generation.	Increased LVEF by 20%	Cyclosporine A	Intravenous	5 min before reperfusion	2022
(158)	Apoptotic Body Membrane-Hybrid Nanocarrier	Targets inflammatory sites via apoptotic body membrane adhesion molecules; releases HAL to initiate heme metabolism, generating anti-inflammatory bilirubin to enhance M2 polarization.	Increased LVEF by 6%	Hexyl-5-aminolevulinate	Intravenous	3 days after ischemia	2022
(159)	Monocyte Membrane-Modified Extracellular Vesicles	Evades immune clearance; Tβ4 activates HIF-1α pathway to promote endothelial migration and angiogenesis.	Increased LVEF by 45%, reduced infarct size by 11%	Thymosin β4	Intravenous	3 consecutive days post-AMI induction	2023
(160)	Polymeric NPs	Suppresses monocyte/macrophage recruitment to infarcted myocardium.	Reduced infarct size by 12%	Pitavastatin	Intravenous	Immediately after reperfusion	2021
(161).	Bimetallic Nanozyme (Cu-TCPP-Mn)	Scavenges ROS; targets ischemic myocardium, suppresses IL-1β/TNF-α, reduces neutrophil/macrophage infiltration, elevates IL-10.	Reduced infarct size by 23.96%, increased LVEF by 25%	–	Intravenous	Immediately after AMI	2023
(142)	Prussian Blue Nanozyme	Scavenges ROS; drives macrophage polarization to M2 phenotype; activates AMPK pathway and inhibits NF-κB pathway.	Reduced infarct size by 21%, increased LVEF by 14%	–	Intravenous	0 day post-AMI, repeated on days 3,7	2025
(162)	CeO_2_ NPs	Scavenges ROS; activates Nrf2/ARE pathway to suppress M1 macrophage polarization.	Increased LVEF by 30%	Nrf2 plasmid	Intravenous	30 min post-AMI, repeated on days 3,5,7	2025
(163)	Mesoporous Silica NPs	Targets CD11b-positive monocytes/neutrophils in infarct zone; enriches NGR1 in damaged myocardium; reduces apoptosis and modulates macrophage phenotype (↓M1, ↑M2).	Reduced infarct size by 19%, increased LVEF by 22%	Notoginsenoside R1	Intravenous	24 h after MI	2022
(164)	Erythrocyte Membrane-Coated Polymeric NPs	Targets berberine to infarcted myocardium; inhibits inflammation and promotes reparative macrophage proliferation.	Increased LVEF by 40%	Berberine	Intravenous	15 min after MI	2023

### 3.4 Tergs-targeting nanoparticle-based drug delivery systems

Tregs within ischemic myocardial tissue can exert protective effects, including anti-apoptotic, anti-inflammatory, and antioxidant actions, which may help reduce left ventricular remodeling (45). The accumulation of Tregs in the ischemic heart is considered an important factor for supporting cardiac repair. Systemic delivery of exogenous Tregs to increase their circulating levels after AMI has been associated with improved cardiomyocyte survival, cardiac function, and overall repair outcomes (98).

In addition to direct Treg infusion, some strategies aim to expand Treg populations locally. Fangyuan Li et al. developed platelet membrane-coated NPs (CsA@PPTK) that target ischemic myocardium. CsA@PPTK can scavenge ROS and increase the Treg population and the M2/M1 macrophage ratio. In high-ROS environments, the PTK component degrades to release CsA, which inhibits mPTP over-opening and may help reduce cardiomyocyte apoptosis, inflammation, and fibrosis (157).

In another approach, Kwon et al. designed liposomal NPs (L-Ag/R) co-loaded with myocardial infarction-associated antigens and rapamycin. After intravenous administration and uptake by DCs, L-Ag/R can induce tolerogenic dendritic cells and promote the generation of antigen-specific Tregs. These Tregs migrate to the infarcted myocardium, potentially enabling more targeted immune tolerance with reduced risk of non-specific systemic immunosuppression compared to polyclonal Tregs. They appear to suppress pro-inflammatory M1 macrophage activity and promote M2 macrophage polarization, which may help mitigate local inflammation, reduce cardiomyocyte apoptosis, and limit fibrosis (165).

Current research in this area often focuses on engineered cell therapies, such as CAR-Tregs, or small-molecule immunomodulators like rapamycin, which can directly expand or activate Tregs (96). In contrast, nanoparticle-mediated targeting of Tregs remains less explored. This may be due to challenges in achieving specificity, as Tregs share surface markers with effector T cells (e.g., CD25, CTLA-4), combined with the generally low infiltration of Tregs into inflammatory MI zones. These factors can limit efficient nanoparticle targeting and accumulation, indicating a need for further investigation (97) (Table 3).

**Table 3:** Tergs-targeting nanoparticle-based drug delivery systems.

Reference	Nanoparticle type	Targeting mechanism	Efficacy endpoints	Therapeutic agent	Administration route	Administration time	Year
(157)	Platelet Membrane-Biomimetic NPs	Scavenges ROS; increases Tregs generation; modulates M2/M1 macrophage ratio; inhibits mPTP over-opening	Reduced infarct size by 8.5%, increased LVEF by 25%	Cyclosporine A	Intravenous	5 min before reperfusion	2022
(165)	Liposomal NPs	Induces tDCs; increases antigen-specific Tregs recruitment; suppresses M1 and promotes M2 macrophages to reduce inflammation	Reduced infarct size by 18%, increased LVEF by 13%	MI tissue lysates (antigen) + Rapamycin	Intravenous	24 h post-AMI	2021

CAR-Treg-NP therapy is being explored for application in cardiovascular diseases. In the context of cardiac fibrosis, lipid NPs encapsulating mRNA encoding FAP-targeting chimeric antigen receptors have been employed to generate transient CAR-T cells *in vivo* through a single intravenous injection. These cells selectively clear activated cardiac fibroblasts, which has been shown to reduce fibrosis and improve cardiac function in mouse models (166). This CAR-Treg-NP strategy represents a potential direction for the precise modulation of immune-mediated cardiovascular pathologies.

### 3.5 Comparative overview of major nanoparticle-based drug delivery systems for AMI therapy

NP-NDDSs represent a promising approach for targeted therapy in AMI. Different classes of NPs offer distinct advantages and limitations. Organic NPs, such as liposomes, polymeric NPs (e.g., PLGA), and micelles, generally exhibit high biocompatibility, tunable drug release profiles, and ease of surface functionalization. However, they may present challenges related to stability, potential drug leakage, and local acidification caused by degradation products (121, 122, 126, 127, 129, 130). Inorganic NPs, including gold (AuNPs) and silica nanoparticles, often provide high physicochemical stability and substantial drug-loading capacity. A potential limitation is their relatively limited biodegradability, which raises considerations about long-term accumulation and possible toxicity (134, 135, 143, 144). Biomimetic NPs, such as cell membrane-coated or exosome-based systems, offer advantages in immune evasion, targeting ability, and biocompatibility. Their development, however, can face challenges related to scalable production and batch-to-batch consistency (18, 20, 142, 148). Overall, organic and biomimetic NPs may have greater clinical potential due to their biodegradable nature and bioinspired properties, whereas inorganic NPs may require further development to address safety profiles. A systematic comparison of key parameters—including drug loading capacity, biocompatibility, targeting accuracy, and translational feasibility—is provided in Table 4 to aid in the rational selection of NP-NDDSs for AMI therapy.

**Table 4:** Comparative summary of nanoparticle-based drug delivery systems for AMI therapy.

Category	Structure/composition	Key advantages	Key limitations	Applications/drug types for AMI	Clinical translation potential/status	Reference
Liposomes	Phospholipid bilayer vesicle, amphiphilic	High biocompatibility; Co-delivery of hydrophilic/hydrophobic drugs; Clinically validated	Stability issues (leakage, aggregation); Rapid clearance by MPS	Gene/drug co-delivery (129); Anti-inflammatories; Nucleic acids (siRNA, miRNA)	High. Multiple FDA-approved products (e.g., Doxil). No AMI-specific approval yet.	(129, 130, 165)
Polymeric NPs (e.g., PLGA)	Biodegradable polymers (e.g., PLA, PGA, PLGA)	Controllable degradation & release; Tunable properties; Excellent biocompatibility	Acidic degradation products may cause local acidification	Widely Used. TLR4 inhibitors (152), PPARγ agonists (146), Senolytics (156), steroids (150), statins (160).	High. PLGA is FDA-approved (e.g., Lupron Depot). Leading candidate for AMI.	(125, 126, 146, 152, 156, 160)
Micelles	Self-assembled amphiphilic molecules, hydrophilic shell/hydrophobic core	Small size (<80 nm) for penetration; High hydrophobic drug loading	Low stability upon dilution; Premature drug release	CCR2 antagonists (153); Hydrophobic anti-inflammatory agents	Moderate. Approved for cancer (e.g., Genexol-PM). Stability needs improvement for AMI.	(120, 121, 153, 155)
Dendrimers	Highly branched, symmetric macromolecules	Multivalent surface; Well-defined size and structure	Potential toxicity; Complex synthesis	Targeted delivery of imaging agents and drugs	Low to moderate. Toxicity and manufacturing challenges. Mostly preclinical.	(128)
Gold NPs (AuNPs)	Metallic gold core, various shapes/sizes	Facile synthesis; Tunable optics; Low toxicity; Biocompatibility	Non-biodegradable; Long-term accumulation concerns (167, 168).	Cardioprotective drugs (133); Photothermal therapy; Imaging	Moderate (for therapy). FDA-approved for photothermal therapy. Long-term safety uncertain.	(131, 133)
Silica NPs (Mesoporous)	Silica framework with porous structure	High surface area; High drug loading; Easily functionalized	Slow biodegradation; Long-term safety not established (135, 168)	Adenosine (134); TCM monomers (163); Nucleic acids; Proteins.	Low to moderate. No FDA-approved drug carriers. Regulatory hurdles due to biodegradability.	(134, 163)
Nanozymes (e.g., Prussian Blue, CeO_2_)	Inorganic crystals with enzyme-like activity	ROS scavenging; Multifunctional; High stability	Long-term biodistribution/clearance unclear	Antioxidant therapy to mitigate oxidative stress (142, 162).	Emerging/Low. Considered new chemical entities (NCEs). Entirely preclinical for AMI.	(142, 143, 161, 162)
Cell Membrane-Coated NPs	Synthetic NP core coated with natural cell membrane	Immune evasion; Inflammatory targeting; Biocompatibility	Complex manufacturing; Batch variability; Scalability issues	siRNA (20, 147), cytokines (18), microRNA (141), small molecule drugs (157).	High (future potential). Promising preclinical results. No clinical trials for AMI yet.	(18, 20, 141, 147, 157, 158, 164)
Extracellular Vesicles/Exosomes	Natural lipid bilayer vesicles with proteins/nucleic acids	Natural targeting; Low immunogenicity; Cross biological barriers	Low yield; Heterogeneity; Difficult drug loading/standardization	Natural cargo (miRNAs, proteins); Engineered drugs/nucleic acids.	Moderate/High (as biologics). Many clinical trials ongoing. Manufacturing is a major hurdle.	(159)
Apolipoprotein-based NPs	Apolipoprotein-phospholipid complexes mimicking HDL	Innate receptor targeting; Cholesterol efflux; Anti-inflammatory	Limited loading for non-lipophilic drugs; Complex composition	Immunomodulation of neutrophils/monocytes (151).	Low (as drug carrier). Clinical trials for lipid management (e.g., CER-001), not AMI drug delivery.	(151)

## 4 Challenges facing nanoparticle drug delivery systems for myocardial infarction therapy

### 4.1 Challenges In clinical translation

Most nanotherapeutics for MI remain at the preclinical stage, having been tested primarily in small animal models such as mice and rats. Among the studies reviewed here, only one included a small-scale clinical trial (151), while all others are still in the preclinical phase. This heavy reliance on animal models represents a major limitation in the development of NP-NDDS for AMI.

Although clinical research on NP-NDDS for AMI is limited, some studies in broader cardiovascular fields have begun to explore clinical applications. For example, Lu Yang et al. conducted a randomized controlled trial using a liposomal NP-NDDS to co-deliver low-dose clopidogrel and aspirin. The study involved 270 patients with coronary artery disease and reported that the liposomal formulation reduced the incidence of major adverse cardiovascular events with a favorable safety profile compared to conventional dual antiplatelet therapy (169).

However, not all clinical trials have yielded positive outcomes. In a study by Fleur M. van der Valk et al., 30 patients with atherosclerosis received liposomal prednisolone. Although the nanoparticles reached macrophages within atherosclerotic plaques, no significant anti-inflammatory effect was observed—possibly due to insufficient drug dosage or the small sample size (170). Another planned trial led by Yan Fang et al. aims to enroll 200 participants to evaluate a self-assembling nanoprobe for detecting fibroblast growth factors (FGFs) for cardiovascular disease screening and treatment assessment (ChiCTR2400089047). Despite considerable preclinical progress, the translation of NP-NDDS into clinical practice remains limited.

The challenges in clinical translation are partly attributable to anatomical, physiological, and pathological differences between species. For instance, the heart rate of mice (500–700 bpm) is much higher than that of humans (60–100 bpm), which affects hemodynamic shear stress and may alter NP circulation time, protein corona formation, and targeting efficiency. Pathologically, post-AMI inflammation in rodents is relatively short, typically resolving within 3–7 days, with fibrotic remodeling often complete within a week. In humans, however, the inflammatory phase can persist for 2–4 weeks, and fibrotic remodeling is more prolonged and complex.

Heavy reliance on small animal models means that key parameters such as targeting efficiency and safety observed in rodents may not reliably predict outcomes in humans. Additionally, clinical patients often exhibit significant individual variability—unlike standardized animal models. Factors such as immune status, age, and comorbidities can further influence NP efficacy and targeting. Therefore, the use of large animal models such as porcine MI models, which more closely mimic human physiology and pathology, is essential. These models allow for better evaluation of NP targeting efficiency, long-term safety (e.g., tracking cardiac function over ≥6 months), and immunogenicity (e.g., anti-NP antibody generation), thereby improving predictive validity.

Another challenge lies in the choice of administration route and the dynamic nature of the inflammatory response following AMI. The administration method significantly affects NP accumulation in the infarcted area, as well as the duration of therapeutic action and overall safety. Direct intramyocardial injection can achieve high local drug concentrations but may cause mechanical injury to vulnerable cardiac tissue and increase the risk of myocardial rupture. Intravenous injection is less invasive but often results in rapid systemic clearance and delayed delivery to the target site. As shown in the study by van der Valk et al., even when NPs reach the lesion, treatment effects may be limited by factors such as insufficient dosing (151).

Moreover, the post-AMI inflammatory response evolves dynamically over time, involving phenotypic shifts in immune cells such as N1/N2 neutrophils and M1/M2 macrophages. This underscores the need for NP systems capable of spatiotemporally controlled drug release. Future research could focus on developing “phase-specific” NPs that adapt to the changing inflammatory microenvironment—from acute injury to repair phases. Strategies may include dual-targeting ligands that respond to both microenvironmental cues and specific cell phenotypes, or biomimetic approaches that leverage the homing behavior of immune cells, such as neutrophil- or macrophage-mimetic NPs.

With advances in computational methods, artificial intelligence (AI)-assisted design has emerged as a potential tool for developing NPs with spatiotemporally controlled release properties. Machine learning (ML) models can analyze high-throughput experimental and computational data to predict NP behavior in complex inflammatory microenvironments, supporting more rational NP design (171). For example, ML can help predict protein corona composition based on protein sequences and NP physicochemical properties (172, 173), which may guide the design of surface ligands to enhance targeting toward specific cell phenotypes (e.g., M1/M2 macrophages) while reducing non-specific uptake. In ligand optimization, AI may aid in identifying dual- or multi-targeting ligands that respond to microenvironmental signals (e.g., pH, ROS) and cell surface receptors, enabling NPs to adapt to stage-specific inflammatory cues. ML models may also analyze relationships between immune cell homing behavior and membrane protein expression, informing the design of biomimetic nanocarriers (174). By integrating multi-omics data with computational modeling, AI-assisted approaches could help design NPs that dynamically adjust their function during disease progression, improve targeting accuracy, and reduce off-target effects.

### 4.2 Regulatory and manufacturing hurdles

While nanoparticles offer potential advantages in medical applications, they also present considerable immunogenic risks. Owing to their surface properties and foreign nature, nanoparticles can activate the immune system, potentially triggering hypersensitivity or inflammatory responses. For instance, gold nanoparticles (AuNPs) used in diagnostic imaging have been associated with allergic reactions such as rashes, swelling, and respiratory distress in some patients (175). Dendrimers and polymeric nanoparticles may activate immune cells while enhancing drug delivery, which could lead to inflammation or allergic reactions (176). Even typically biocompatible carriers such as liposomes have been reported to induce immune responses under high-dose or long-term administration (177, 178). These observations highlight the importance of prioritizing endogenous degradable materials (e.g., hyaluronic acid, chitosan) or biomimetic materials (e.g., exosomes, cell membrane vesicles) to help minimize long-term toxicity risks. It is also considered essential to expand long-term safety assessments to include large animal models.

The metabolic pathways, accumulation effects, and potential chronic health impacts of nanoparticles in humans remain inadequately understood, partly due to a lack of large-scale clinical studies (179). Although two clinical studies in cardiovascular disease using lipid nanoparticles reported no major safety concerns, their sample sizes were limited (169, 170). Animal studies have indicated that certain inorganic nanoparticles can accumulate in organs such as the liver, lungs, and kidneys, where they may induce oxidative stress, inflammation, or other adverse effects (180). Additionally, the environmental behavior of nanoparticles warrants attention: they may enter soil and water systems through medical waste or emissions, with the potential to bioaccumulate and move through the food chain, posing possible risks to ecosystems and public health (179, 181). Therefore, advancing the development of biodegradable nanomaterials and strengthening regulatory oversight of NPs production and use are important priorities.

These scientific uncertainties further complicate the regulatory evaluation of nanomedicine products. Regulatory agencies such as the U.S. FDA and the European EMA currently rely largely on frameworks designed for conventional drugs, which may not fully address the specific complexities of nanomaterials, including batch-to-batch variability, bioequivalence assessment, and dynamic release behaviors (182–184). The manufacturing of nanomedicines requires precise control over critical parameters such as particle size distribution, surface charge, drug encapsulation efficiency, and release kinetics, which can vary between batches and pose challenges to product consistency and quality (185). Adherence to Good Manufacturing Practice (GMP) and the establishment of a comprehensive quality control system—covering raw materials, production processes, and final products—are essential to ensure reproducible quality during scale-up from laboratory research to commercial production (185). Some nanomedicines, including Doxil and Abraxane, have encountered delays or obstacles in approval related to stability, toxicity, or manufacturing consistency issues (184). Moreover, the absence of harmonized international regulatory standards may limit the global accessibility and equitable distribution of nanomedicines (186, 187). To support the responsible development of nanomedicine, there is a need to establish transnational collaborative mechanisms and develop unified global evaluation standards specifically addressing the safety, efficacy, and environmental impact of nanomaterials.

## 5 Conclusion

NP-NDDSs may offer potential benefits for advancing the treatment of AMI through targeted modulation of the inflammatory immune response. As discussed, these systems have been shown in preclinical studies to enhance the precision of therapeutic delivery to pivotal immune cells, suggesting potential for reducing tissue damage and supporting repair processes.

The path to clinical application, however, involves considerable challenges. A central issue is the evolving inflammatory landscape after AMI, which requires drug delivery systems that can adapt over time. Future efforts should focus on creating versatile platforms that can sequentially modulate different phases of the immune response. Incorporating biomimetic designs, such as cell membrane coatings, may improve targeting specificity, while artificial intelligence could aid in optimizing nanoparticle properties for desired *in vivo* performance. Additionally, robust validation in physiologically relevant large animal models is essential to reliably assess therapeutic benefits and safety profiles. Translational progress will also depend on overcoming hurdles in manufacturing consistency, long-term biosafety, and navigating evolving regulatory frameworks.

In conclusion, while NP-NDDSs represent a **promising approach** to AMI therapy, their successful translation will depend on addressing these interrelated biological and technical challenges.
